# Diagnosis and management of primary aldosteronism

**DOI:** 10.1590/2359-3997000000274

**Published:** 2022-05-01

**Authors:** Leticia A. P. Vilela, Madson Q. Almeida

**Affiliations:** 1 Hospital das Clínicas Faculdade de Medicina Universidade de São Paulo São Paulo SP Brasil Unidade de Suprarrenal, Endocrinologia do Desenvolvimento, Laboratório de Hormônios e Genética Molecular – LIM42, Divisão de Endocrinologia e Metabologia, Hospital das Clínicas, Faculdade de Medicina da Universidade de São Paulo (HCFMUSP), São Paulo, SP, Brasil; 2 Instituto do Câncer do Estado de São Paulo FM USP São Paulo SP Brasil Instituto do Câncer do Estado de São Paulo (Icesp), FMUSP, São Paulo, SP, Brasil

**Keywords:** Primary aldosteronism, resistant hypertension, diagnosis, aldosterone, renin

## Abstract

Primary aldosteronism (PA) is the most common form of secondary hypertension (HTN), with an estimated prevalence of 4% of hypertensive patients in primary care and around 10% of referred patients. Patients with PA have higher cardiovascular morbidity and mortality than age- and sex-matched patients with essential HTN and the same degree of blood pressure elevation. PA is characterized by an autonomous aldosterone production causing sodium retention, plasma renin supression, HTN, cardiovascular damage, and increased potassium excretion, leading to variable degrees of hypokalemia. Aldosterone-producing adenomas (APAs) account for around 40% and idiopathic hyperaldosteronism for around 60% of PA cases. The aldosterone-to-renin ratio is the most sensitive screening test for PA. There are several confirmatory tests and the current literature does not identify a “gold standard” confirmatory test for PA. In our institution, we recommend starting case confirmation with the furosemide test. After case confirmation, all patients with PA should undergo adrenal CT as the initial study in subtype testing to exclude adrenocortical carcinoma. Bilateral adrenal vein sampling (AVS) is the gold standard method to define the PA subtype, but it is not indicated in all cases. An experienced radiologist must perform AVS. Unilateral laparoscopic adrenalectomy is the preferential treatment for patients with APAs, and bilateral hyperplasia should be treated with mineralocorticoid antagonist (spironolactone or eplerenone). Cardiovascular morbidity caused by aldosterone excess can be decreased by either unilateral adrenalectomy or mineralocorticoid antagonist. In this review, we address the most relevant issues regarding PA screening, case confirmation, subtype classification, and treatment.

Hypertension (HTN) affects between 10 to 40% of the general population and is the leading risk fator for premature death in the world (1,2). Robust experimental and clinical evidence implicates mineralocorticoids in the pathogenesis of HTN (3). Most monogenic forms of HTN in humans can be associated to defects in renal sodium balance (4). Several studies demonstrated that elevated aldosterone levels are predictors of adverse outcome in HTN (5), heart failure (6,7), myocardial infarction (8), and renal insufficiency (9).

Primary aldosteronism (PA) is the most common form of secondary HTN with an estimated prevalence of 4% of hypertensive patients in primary care and around 10% of referred patients (10,11). PA is particularly common in patients with resistant HTN, with a prevalence of 14 to 21% (12,13). Resistant HTN is defined as office, or clinic, systolic blood pressure (BP) of ≥ 140 mmHg, diastolic BP of ≥ 90 mmHg, or an elevation of both, on at least 3 antihypertensive medications from different drug classes, preferably including a diuretic (14). PA is the most common curable form of HTN. Because of the adverse cardiovascular effects of excess aldosterone that are independent of high BP levels, patients with PA have higher cardiovascular morbidity and mortality than age- and sex-matched patients with essential HTN and the same degree of BP elevation (15,16) (Table 1).

**Table 1:** Cardiovascular and metabolic complications in primary aldosteronism (PA) compared to essential hypertension (EH)

	PA (%)	EH (%)	*p*
**Cardiovascular events**			
Atrial fibrilation (17)	3.9	1.1	0.001
Coronary artery disease (17)	5.7	2.8	0.03
Heart failure (17)	4.1	1.2	0.003
Nonfatal myocardial infarction (17)	4.4	1.7	0.01
Stroke (18)	7.4	3.5	0.006
**Metabolic alterations**			
Metabolic syndrome (19)	41.1	29.6	0.05
Abnormal glucose metabolism (20)^#^	22.4	16.8	0.04

^#^ Meta analysis.

PA is characterized by an autonomous aldosterone production which is inappropriately high for sodium state and is not regulated by angiotensina II or plasma potassium concentrations. This autonomous aldosterone production causes sodium retention, plasma renin supression, HTN, cardiovascular damage, and increased potassium excretion, leading to variable degree of hypokalemia (21). In the largest study of PA prevalence, hypokalemia was identified in 48% of aldosterone-producing adenomas (aldosteronomas) and in 17% of idiopathic hyperaldosteronism (bilateral adrenal hyperplasia) (22). The frequency of PA subtypes and hypokalemia in different cohorts depends on whether PA is routinely screened among hypertensive patients and if adrenal vein sampling (AVS) is available in the specialized center. In general, aldosterone-producing adenomas (APAs) account for around 40% and idiopathic hyperaldosteronism for around 60% of PA cases. APAs are small benign tumors (1-3 cm) originating from the glomerulosa zone, but in few cases can be smaller than 1 cm and diagnosed only if the AVS shows a lateralized aldosterone production. If a patient with PA has an adrenal tumor larger than 4 cm, we should consider the rare possibility of an aldosterone-producing adrenal carcinoma. Other rare causes of PA, accounting for less than 1%, are unilateral adrenal hyperplasia and familial PA, which is discussed elsewhere in this review. In our cohort of 104 PA cases from the Clinics Hospital of Sao Paulo University Medical School, aldosteronomas were diagnosed in 79% and idiopathic hyperaldosteronism in 20% of our cases, suggesting that idiopathic hyperaldosteronism is probably underdiagnosed at our institution. Familial PA type I (glucocorticoid remediable form of PA) was diagnosed in a single patient. Hypokalemia was identified in 79% of patients with aldosteronomas and in 68% of those with idiopathic hyperaldosteronism. The case detection of PA is recommended in the conditions listed in Table 2, as addressed in the 2016 Endocrine Society Clinical Practice Guideline of PA (23).

**Table 2:** Recommendations for primary aldosteronism (PA) screening in clinical setting

Hypertension and spontaneous or diuretic-induced hypokalemia
Hypertension and adrenal incidentaloma
BP above 150/100 on three separate measurements obtained on different days
Hypertension (BP ≥ 140/90) resistant to three conventional antihypertensive drugs (preferably including a diuretic)
Controlled BP (< 140/90) on four or more antihypertensive drugs
Hypertension and sleep apnea
Hypertension and a family history of early onset hypertension or cerebrovascular accident at a young age (< 40 years)
All hypertensive first-degree relatives of patients with PA

BP: blood pressure.

## SCREENING AND CASE CONFIRMATION

**Figure 1:**
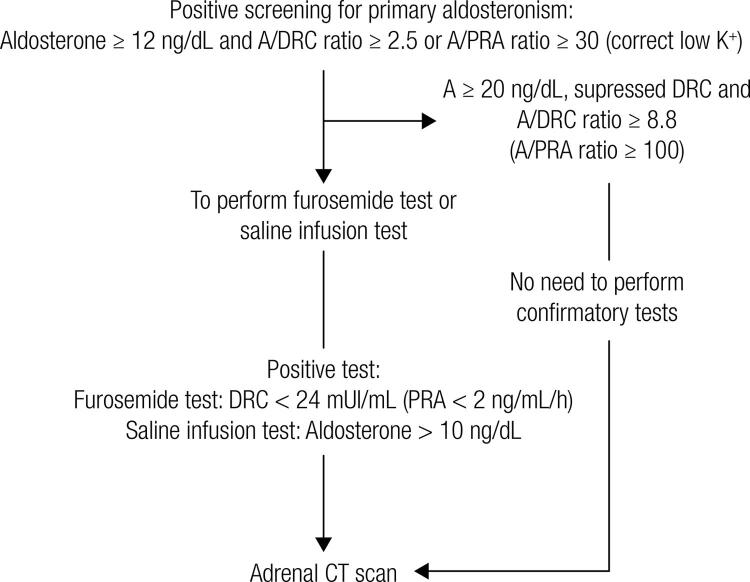
Algorithm for the detection and confirmation of primary aldosteronism. A: aldosterone; DRC: direct renin concentration; PRA: plasma renin activity; CT: computed tomography.

The aldosterone-to-renin ratio is the most sensitive test that screens for PA. Immunometric assays can be employed to measure renin either by testing for plasma renin activity (PRA) or for direct renin concentration (DRC). Currently, most comercial kits measure direct renin concentration; however, the aldosterone-to-renin ratios used in PA screening were determined using PRA. Because the aldosterone-to-renin ratio is more dependent on renin, assays should be sensitive enough to detect PRA levels of 0.2-0.3 ng/mL/h (or DRC of 2 mU/L). When measuring plasma aldosterone (A) in ng/dL and PRA in ng/mL/h, the most commonly adopted A/PRA cut-off for PA screening is 30 (21). In the presence of low renin levels, the aldosterone-to-renin ratio may be elevated even when aldosterone is not high. Then, a minimum aldosterone concentration of 15 ng/dL has been proposed as part of the screening criteria. In our institution, 6% of PA patients had aldosterone levels between 12.5 and 15 ng/dL. Because of that, we use the minimum aldosterone level of 12.5 ng/dL to proceed with PA investigation (Figure 1). In a recently introduced and already commonly used automated DRC assay (DiaSorin, LIAISON XL instrument), we can use the conversion factor of 12 (DRC/12 = PRA) to calculate the A/PRA ratio (21). A recent study validated this automated chemiluminescent assay for DRC and aldosterone. Using DRC (mU/L), the A/DRC ratio of 2.1 had a sensitivity of 92%, a specificity of 92%, and negative predictive value of 99% to PA diagnosis. When using the A/DRC of 3.3, sensitivity was 84% and specificity was 96% for PA diagnosis (24). Although we need more studies to validate the aldosterone-to-renin ratio using DRC, it seems that A/DRC between 2 and 3 should be equivalent to A/PRA ratio of 30.

In order to increase sensitivity, aldosterone and renin samples should be collected in the morning after patients have been out of bed for at least 2 hours, and usually after they have been seated for 5-15 minutes. Ideally, patients should have unrestricted dietary salt intake before testing and should be potassium-replete (21). It should be emphasized that mineralocorticoid antagonists (spironolactone or eplerenone) and other types of diuretics should be withdrawn for at least 4 weeks before testing. When it is not clinically possible to withdraw mineralocorticoid antagonists or diuretics, suppressed renin levels associated with high aldosterone levels strongly suggest a PA diagnosis.

In many cases (excluding mineralocorticoid antagonist or diuretic treatment), the aldosterone-to-renin ratio can be confidently interpreted despite the effect of continued medications, thus avoiding delay and allowing the patient to proceed directly to confirmatory testing. Very often, a washout of all interfering antihypertensive medications is not feasible in patients with severe HTN. Then, the aldosterone-to-renin ratio should be interpreted in light of the potential confounding factors (Table 3) (21). In premenopausal ovulating women, false positives can occur during the luteal phase, but only if renin is measured as DRC. Similarly, the use of oral contraceptives is associated with a false positive screening for PA, but only if renin is measured as DRC (25). Then, if premenopausal women have a positive screening for PA, we can repeat the test without oral contraceptives (if is the case) or during the follicular phase, or even proceed with confirmatory testing.

**Table 3:** Factors that may interfere with screening for primary aldosteronism

	Aldosterone	DRC	Aldosterone/DRC
β-Adrenergic blockers	↓	↓	↑(FP)
Central agonists (clonidine, α-methyldopa)	↓	↓	↑(FP)
Diuretics	→↑	↑	↓(FN)
Ca^2^ blockers (DHPs)	→↑	↑	↓(FN)
ACE inhibitors, ARBs	↓	↑	↓(FN)
Advancing age	↓	↓	↑(FP)
Premenopausal women (vs. Male)	→↑	↓	↑(FP)

DRC: direct renin concentration; FP: false positive; FN: false negative; ARBs: angiotensin II type 1 receptor blockers; DHP: dihydropyridines.

In the Endocrine Division of the Clinics Hospital of University of Sao Paulo Medical School, we follow the algorithm for detection and confirmation of PA shown in Figure 1. First, potassium levels should be normal to adequately interpret the aldosterone-to-renin ratio. If a patient has aldosterone levels ≥ 12.5 ng/dL and A/PRA (ng/mL/h) ratio ≥ 30 or A/DRC (mU/L) ratio ≥ 2.5, the PA screening is considered positive. In our cohort, all PA patients had the A/PRA ratio above 30 or A/DRC ratio above 2.5 when DRC was determined.

As mentioned before, we always recommend the withdrawn of mineralocorticoid antagonists and diuretics for at least 4 weeks when clinically feasible. Otherwise, we perform the PA screening with all other antihypertensive medications. If aldosterone levels are < 20 ng/dL and PRA or DRC are not suppressed (but within the lower normal range), we recommend the replacement of the antihypertensive medications with verapamil, hydralazine, and α-1 blockers (doxazosin or prazosin). Clonidine can be added if a fourth medication is needed.

- If a patient has aldosterone levels ≥ 20 ng/dL and suppressed PRA or DRC levels, there is no need for further confirmatory testing and we can proceed with a computed tomography (CT) scan to investigate PA etiology (26). In other cases, we perform confirmatory testing to exclude false positives (Figure 1). There are several confirmatory tests and the current literature does not identify a “gold standard” confirmatory test for PA (21). Specialized centers choose their preferred confirmatory test based on their own experience. We suggest starting case confirmation with the furosemide test. In our experience, the furosemide test had an accuracy higher than 90% in diagnosing PA. If the furosemide test is not conclusive and the patient doesn’t have contraindication for sodium loading, we perform the saline infusion test. The main problem with the saline infusion test is the volume overload in a short period performed in patients with severe/resistant HTN or congestive heart failure. Currently, we only use the captopril test as a first option in patients with very impaired renal function, because of its too low reproducibility. Before performing any confirmatory test, we should check to see if potassium levels are normal and should correct hypokalemia. Below is detailed description of the three confirmatory tests mentioned above:
- **Furosemide test:** Patients receive furosemide 40 mg iv and stay in an upright position for 2h, starting at 8-9.30 AM. Blood samples for PRA or DRC, aldosterone, and potassium are drawn at time zero and after 2 h. PRA < 2 ng/mL/h (or DRC < 24 mU/L) confirms the PA diagnosis (26). Although patients with essential hypertension can present low renin levels, plasma renin activity (PRA) increases above 2 ng/mL/h after furosemide injection.
- **Saline infusion test:** Patients stay in the recumbent position for at least 1 h before and during the infusion of 2L of 0.9% saline iv over 4h, starting at 8-9.30 AM. Blood samples for aldosterone, PRA or DRC, and potassium are drawn at time zero and after 4 h. Aldosterone levels > 10 ng/dL confirm the diagnosis of PA, and aldosterone < 5 ng/dL excludes the diagnosis. Aldosterone levels between 5 and 10 ng/dL are considered indeterminate, although a cutoff of 6.8 ng/dL has been found to have the best trade-off between sensitivity and specificity (27).
- **Captopril test:** Patients receive 50 mg of captopril orally after sitting or standing for at least 1 h. Blood samples are drawn for measurement of PRA or DRC, plasma aldosterone, and cortisol at time zero and at 1h and 2h after captopril, with the patient remaining seated during this period. Plasma aldosterone is normally suppressed by captopril (> 30%). In patients with PA, aldosterone level remains elevated and renin remains suppressed (26). APAs can be abnormally regulated by ACTH. Then, if cortisol levels decrease during the test, we should diminish the percentage of cortisol variation from aldosterone variation to analyze only the captopril effect.

## SUBTYPE CLASSIFICATION

**Figure 2:**
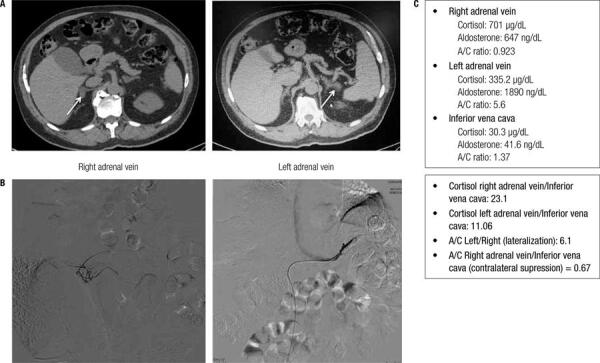
A male patient, 69 years, with resistent hypertension for 30 years, diagnosed with PA. Hormonal data: aldosterone (A) = 24.4 ng/dL; DRC < 1.6 mU/mL (PRA 0.13); A/DRC ratio = 15.25; A/PRA ratio = 188. (A) Adrenal CT showing a 1.8 cm nodule at right adrenal gland and a 1.2 cm nodule at left adrenal gland. (B) Fluoroscopic imaging from AVS. (C) Analysis of AVS sampling showed lateralization of aldosterone production to the left side. Then, this patient had an adrenal incidentaloma on the right side and an aldosterone-producing adenoma on the left side. The patient underwent left adrenal adrenalectomy and had biochemical cure of PA.

All patients with PA should undergo adrenal CT as the initial study in subtype testing to exclude adrenocortical carcinoma. Magnetic resonance imaging has no advantage over CT in subtype evaluation of PA (21). Proper distinction between APAs and bilateral hyperplasia is crucial, because the former is treated by adrenalectomy and the latter by mineralocorticoid receptor antagonists. For the diagnosis of these two subtypes, adrenal CT scan or bilateral adrenal vein sampling (AVS) is used. Adrenal CT has several limitations, because its accuracy for diagnosing APAs is limited. CT scan can reveal normal-appearing adrenals, but the patient can have a very small APA below the detection limit of CT. Moreover, nonfunctioning unilateral adrenal adenomas are not uncommon, especially in older patients (> age 40 years), and an APA can be incorrectly diagnosed in a patient with bilateral adrenal hyperplasia and normal-appearing adrenal (28). In a different situation, bilateral adrenal nodules might be interpreted as bilateral hyperplasia on the basis of CT findings, but the patient can have an APA and an adrenal incidentaloma in the contralateral side (Figure 2).

The 2016 Endocrine Society Clinical Practice Guideline of PA recommends not to perform AVS in younger patients (< 35 years) with spontaneous hypokalemia, marked aldosterone excess, and unilateral adrenal lesions with radiological features consistent with a cortical adenoma on adrenal CT scan (21). In this situation, the probability of an adrenal incidentaloma is very low. At our institution, we don’t consider the age of PA diagnosis to indicate AVS but the age of HTN diagnosis, because the median time of HTN before PA diagnosis is 14 years. In other words, most of patients with APA had been diagnosed with HTN before 35 or 40 years, but PA was only diagnosed after 40 years. This trend reflects the fact that PA screening is very delayed in our country. Based on this observation, we do not recommend AVS in patients with severe PA (A ≥ 20 ng/dL, hypokalemia and suppressed DRC or PRA) with HTN diagnosed before 40 years and unilateral adrenal lesion (> 1cm) and normal contralateral adrenal on adrenal CT scan.

In a recent randomised controlled trial (SPARTACUS TRIAL), patients with PA were randomly assigned to undergo either adrenal CT or AVS to determine the presence of APA (with subsequent treatment consisting of adrenalectomy) or bilateral adrenal hyperplasia (subsequent treatment with mineralocorticoid receptor antagonists) (29). Of the 184 patients that completed follow-up, 92 received CT-based treatment (46 adrenalectomy and 46 mineralocorticoid receptor antagonist) and 92 received AVS-based treatment (46 adrenalectomy and 46 mineralocorticoid receptor antagonist). The persistence of PA was higher in patients who received CT-based treatment (20%) compared to patients who received AVS-based treatment (11%), but this difference was not statistically significant (29). This important trial showed that AVS should not be recommended for all PA patients, but it did not rule out the importance of AVS in PA subtype classification, particularly in cases with bilateral lesions or normal-appearing adrenal. Although the SPARTACUS trial is the first randomized study to compare CT- *vs.* AVS-based approaches, we need to reanalyze this issue in larger cohorts.

AVS should be performed under a condition of suppressed renin, because increased renin levels by diuretics or mineralocorticoid antagonists can lead to the stimulation of the contralateral adrenal to an APA, and unilateral PA might be misclassified as bilateral. Therefore, it is important to check if DRC or PRA is suppressed before performing AVS. An experienced radiologist is required to perform AVS. AVS includes catheterization and collecting blood samples from the right and left adrenal veins and from the inferior vena cava to measure cortisol and aldosterone. The right adrenal vein may be especially difficult to catheterize because it is short and enters the inferior vena cava at an acute angle (30). We recently started to measure serum cortisol during the AVS procedure to evaluate the successful catheterization of the right adrenal vein. Currently, our rate of successful catheterization is 80%. This strategy has been previously employed to improve successful catheterization (31).

We perform AVS under cosyntropin infusion with sequential bilateral AVS. The use of continuous cosyntropin infusion during AVS minimizes stress-induced fluctuations in aldosterone secretion (since aldosterone can be regulated by ACTH in APAs). In addition, the cosyntropin infusion maximizes the gradient of cortisol from adrenal vein to inferior vena cava, confirming the successful sampling of the adrenal vein (32). To determine the aldosterone lateralization and contralateral suppression, we use the cortisol-corrected aldosterone ratio (aldosterone divided by cortisol level in its respective vein; A/C) to correct for dilution effects. To evaluate if the nondominant adrenal (not oversecreting aldosterone) is suppressed, we calculate the contralateral suppression (defined as the A/C ratio from the low-side to inferior vena cava). See the AVS protocol in the Clinics Hospital of Sao Paulo University Medical School below: 1) Dilute cosyntropin (250 µg) in 250 mL of saline 0.9% and start 50 mL/h (30 min before AVS); 2) Collect blood for aldosterone and cortisol measurements from right and left adrenal veins and from inferior vena cava; 3) Determine cortisol ratio between adrenal veins and the periphery (if the ratio is > 5 on each side, the catheterization was successful); 4) Determine A/C ratio to evaluate lateralization. We should calculate the A/C ratio from high-side to low-side. A ratio of more than 4:1 indicates unilateral aldosterone excess. A ratio of less than 3:1 suggests bilateral aldosterone hypersecretion. With these cutoffs, AVS for detecting unilateral aldosterone hypersecretion has a sensitivity of 95% and specificity of 100%; 5) If the lateralization ratio is between 3:1 and 4:1, the AVS is undetermined. But if contralateral suppression is < 0.67, it suggests a lateralization to the higher side (33). In Figure 2, we demonstrate a clinical case in which AVS was recommended and very useful in the subtype definition.

If a patient with PA has an APA ≥ 2.5 cm or macronodular bilateral hyperplasia, screening for subclinical Cushing is recommended: 1 mg dexamethasone suppression test, ACTH, 24h urinary free cortisol, salivar cortisol and dehydroepiandrosterone sulfate.

## FAMILIAL PRIMARY ALDOSTERONISM

In patients with an onset of confirmed PA earlier than 20 years of age, and in those who have a family history of PA or stroke at a young age (< 40 years), the Endocrine Society Clinical Practice Guideline of PA suggests genetic testing for familial PA type I (glucocorticoid remediable aldosteronism) and type III (caused by *KCNJ5* germline mutations) (21) (Table 4).

**Table 4:** Familial forms of primary aldosteronism

	Type I	Type III	Type III
Cause	Hybrid *CYP11B1/CYP11B2*	Unknown	Germline KCNJ5
Transmission	Autosomal dominant	Autosomal dominant	Autosomal dominant
Genetic diagnosis	Long PCR	No	*KCNJ5* sequencing
Hypertension onset	Very often < 20 years	Adulthood	Very often < 10 years
Hypertension severity	Severe to resistant hypertension (normal BP is rare)	Stage 1 to resistant hypertension (normal BP is not often)	Stage 3 to resistant hypertension
Hypokalemia	Rare	Not often	Very often
Aldosterone after dexamethasone	< 4 ng/dL	> 4 ng/dL	> 4 ng/dL
Adrenal CT	Normal	Unilateral or bilateral lesions	Bilateral macronodular hyperplasia
Treatment	Dexamethasone or mineralocorticoid antagonist	Unilateral adrenalectomy or mineralocorticoid antagonist	Bilateral adrenalectomy or mineralocorticoid antagonist

BP: blood pressure. CT: computed tomography.

Familial PA type I is caused by an unequal crossover between the genes *CYP11B1* (which encodes steroid 11α-hydroxylase) and *CYP11B2* (which encodes aldosterone synthase) (34,35). The resulting hybrid gene encodes an enzyme chimera with aldosterone synthase activity that is expressed in the adrenal zona fasciculata under control of ACTH instead of angiotensin II. Patients with familial PA type I show suppressed plasma aldosterone levels (< 4 ng/dL) after dexamethasone (0.5 mg every 6h during 48 or 72h) (36). Familial PA type I is treated with dexamethasone in adults (0.125-0.25 mg/d). If additional drugs are necessary to control BP or normalize renin levels, mineralocorticoid antagonist can be added (37,38).

Familial PA type II is clinically and biochemically indistinguishable from sporadic forms. Prevalence was reported to be as high as 6% in a large population with PA (39). Familial PA type II is diagnosed when at least two first-degree members of the same family have confirmed PA (APA or bilateral hyperplasia). The molecular basis of FH-II is still unknown, but genetic analyses demonstrated a link with chromosome 7p22 (40).

Familial PA type III is caused by mutations in the *KCNJ5* gene encoding the potassium channel Kir 3.4, resulting in increased sodium conductance and cell depolarization (41). Familial PA type III is characterized by severe HTN in early childhood associated with PA, hypokalemia, and macronodular bilateral hyperplasia (42). Because of the HTN severity, bilateral adrenalectomy may be needed to control BP (36).

## TREATMENT

Cardiovascular morbidity caused by aldosterone excess can be decreased by either unilateral adrenalectomy or mineralocorticoid antagonist (43). Overall reduction of left ventricular mass has been demonstrated to be similar in unilateral adrenalectomy or mineralocorticoid antagonist treatment at the end of a 6.4 year follow-up (44). However, a study comparing both treatments in terms of cardiovascular mortality remains to be conducted.

Unilateral laparoscopic adrenalectomy is indicated for patients with APAs. If the patient with an APA is unable or unwilling to undergo surgery, medical treatment including a mineralocorticoid antagonist is recommended. Before unilateral adrenalectomy, the patient should be treated with mineralocorticoid antagonist in order to normalize potassium levels and renin levels, avoiding hyporeninemic hypoaldosteronism in the postoperative period. In addition, we should measure sodium, potassium, aldosterone, and renin levels in the first week after surgery to monitor treatment response. After unilateral adrenalectomy, HTN is cured in about 50% (range of 35-80%) of patients with APA (21). Primary HTN in first-degree relatives and a longer duration of HTN before diagnosing PA are associated with low rates of HTN cure after unilateral adrenalectomy (45,46).

Bilateral hyperplasia should be treated with mineralocorticoid antagonist (spironolactone or eplerenone). In Brazil, only spironolactone is available for treatment. Spironolactone (50-400 mg/d) has been the agent of choice in the medical treatment of PA, reducing BP levels as well as the need for antihypertensive drugs (47). The starting dose for spironolactone is 50 mg in a single dose. The dose should be increased by 50 mg each 3-4 weeks. During treatment, we aim to control blood pressure and to normalize potassium and renin levels.

Since spironolactone antagonizes androgen receptor and inhibits androgen production, it promotes dose-dependent gynecomastia and loss of libido in men. In females, spironolactone can cause menstrual irregularities and breast tenderness and enlargement (48). To avoid or decrease side effects, amiloride or a small dose of thiazide diuretic can be used to avoid a higher dose of spironolactone. In patients with stage III chronic kidney disease or in older patients, mineralocorticoid antagonist should be administered with caution because of the risk of hyperkalemia and worsening renal function.

Eplerenone is a selective mineralocorticoid antagonist without antiandrogen effects and, therefore, is less associated with endocrine side effects and given twice daily (49). Despite its better tolerability, eplerenone has a higher cost and can be less effective than spironolactone to lower BP in the medical treatment of PA (50). In the next years, it is likely that new mineralocorticoid antagonists and selective aldosterone synthase inhibitors will be available to treat PA (21).
